# Altered Growth and Envelope Properties of Polylysogens Containing Bacteriophage Lambda *N^−^c*I*^−^* Prophages

**DOI:** 10.3390/ijms21051667

**Published:** 2020-02-28

**Authors:** Sailen Barik, Nitai C. Mandal

**Affiliations:** Department of Biochemistry, Bose Institute, Kolkata-700009, India; mandalnc2003@yahoo.com

**Keywords:** bacteriophage lambda, polylysogen, membrane, Gram-negative, lipopolysaccharide, lysogenic conversion

## Abstract

The bacterial virus lambda (λ) is a temperate bacteriophage that can lysogenize host *Escherichia coli* (*E. coli*) cells. Lysogeny requires λ repressor, the *c*I gene product, which shuts off transcription of the phage genome. The λ N protein, in contrast, is a transcriptional antiterminator, required for expression of the terminator-distal genes, and thus, λ N mutants are growth-defective. When *E. coli* is infected with a λ double mutant that is defective in both N and cI (i.e., λ*N^-^c*I*^-^*), at high multiplicities of 50 or more, it forms polylysogens that contain 20–30 copies of the λ*N^-^c*I*^-^* genome integrated in the *E. coli* chromosome. Early studies revealed that the polylysogens underwent “conversion” to long filamentous cells that form tiny colonies on agar. Here, we report a large set of altered biochemical properties associated with this conversion, documenting an overall degeneration of the bacterial envelope. These properties reverted back to those of nonlysogenic *E. coli* as the metastable polylysogen spontaneously lost the λ*N^-^c*I*^-^* genomes, suggesting that conversion is a direct result of the multiple copies of the prophage. Preliminary attempts to identify lambda genes that may be responsible for conversion ruled out several candidates, implicating a potentially novel lambda function that awaits further studies.

## 1. Introduction

One of the earliest studied bacteriophages that contributed extensively to our knowledge of virology and molecular biology [1,2,3], coliphage λ has been well known for its two modes of propagation, lytic and lysogenic, after infection of sensitive *E. coli* host. In the lytic mode, the phage DNA undergoes autonomous replication, following temporally controlled transcription and translation of viral early, delayed early, and late genes, ultimately producing a burst of matured phages that come out by lysing the cell [4]. In the lysogenic mode, however, the phage DNA undergoes site-specific covalent attachment (via the attachment site, abbreviated as *att*, of the phage DNA) with the host DNA, between gal and bio regions, and then continues to replicate passively as part of the host chromosome [5]. The phage genome, remaining in such a dormant state, is called a prophage. The choice between these two modes is dependent on positive and negative control circuits very early in the phage life cycle [4,6,7].

**Figure 1:**
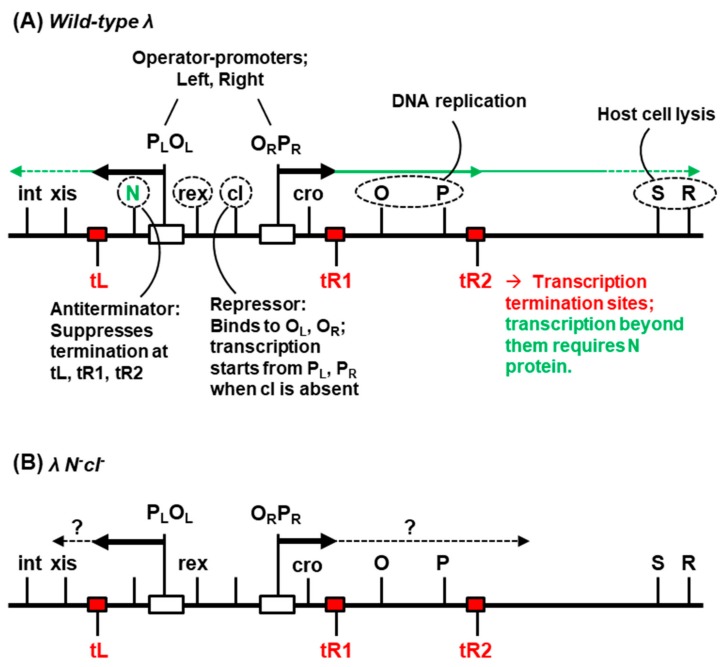
Selected gene and sites in the phage λ genome [2]. Transcription of λ DNA uses host RNA polymerase and is regulated negatively by the transcriptional repressor, cI, and positively by the antiterminator protein, N. In the lytic cycle (**A**), the operators (*O*_L_, *O*_R_) are unoccupied by cI, and leftward and rightward transcription start from the respective promoters, *P*_L_ and *P*_R_. If N is unavailable, ~90% of the transcription [8] initiated from P_L_ and 60% of that initiated from *P*_R_ stops at the termination sites, *t*L and *tR*1, respectively, while the 40% that passes through *tR*1 completely stops at *tR*2 (shown in red). In the presence of N protein, the RNA polymerase is modified so as to ignore the termination sites, which allows transcription to proceed downstream (green arrows). Thus, λ*N*^-^ mutant cannot grow productively. The λ*N*^-^*c*I^-^ double mutant presents a special combination in that it forms the unique polylysogen that is studied in the paper. In this state (**B**), transcription is initiated from both *P*_L_ and *P*_R_, since the *c*I repressor is inactive, but the bulk of the transcription fails to pass the terminators as described above. Several other regulatory genes with accessory functions are not shown for simplicity. Gene names are not italicized in this figure for the sake of simplicity.

The double-stranded DNA genome of λ contains about 40 protein-coding genes and 15 cis-acting regulatory sites [2]. The relevant portion of the genome, encompassing the transcriptional control region, is shown in Figure 1. 

To summarize, the expression of the genes is transcriptionally regulated by several operons, both early and late [4]. For lytic growth, expressions of both the operons are necessary, and they are temporally controlled such that the optimum expression of each gene takes place at the proper time in proper amounts [6,7]. Several transcription termination sites that precede the late operons prevent premature transcription of the late genes, which code mainly for the virion structural proteins and the lysis proteins, S and R that bring about host cell lysis. The termination sites are specifically overcome with the help of the lambda antiterminator protein N, which makes the RNA polymerase termination-resistant, making the lytic cycle possible. The integrase (*int*) gene is located downstream of one such terminator, *t*L, and its expression, therefore, also requires N. The lysogenic state, in contrast, is attained in two stages: establishment, and maintenance. Briefly, early synthesis of the integrase (*int*) promotes site-specific integration of the phage genome with the bacterial genome. As the expression of *int* gene requires N, the latter is required for the establishment of lysogeny as well. It should be noted here that the termination of transcription is somewhat leaky (i.e., even in the absence of N, RNA polymerase reads through the termination sites at a low frequency [8]), which may not be enough for functionally optimal expression of all the downstream genes.

Expression of the transcriptional repressor, cI, is facilitated by the accessory proteins cII and cIII (not shown in Figure 1) through diverse mechanisms [4]. Once sufficient quantities of cI are made, it represses transcription initiation from the early left and right promoters (*P*_L_, *P*_R_), by binding to the corresponding operator sites (*O*_L_, *O*_R_). The cro protein also acts as a transcriptional repressor but exerts a fine-tune control on transcription initiation from these promoters at optimal times so as to promote the lytic growth over lysogenic. The *int*, *xis*, and *rex* genes (Figure 1) are not essential for lytic growth of lambda; int is required for integration of the prophage genome into the *E. coli* genome for establishing lysogeny as well as for the excision of the prophage, while xis is required for excision only, and rex for excluding the growth of T4 phage *r*II mutant [9,10]. The *rex* and *c*I genes are co-transcribed from an independent, shared promoter (known as *prm*, which stands for ‘promoter for repressor maintenance’; not shown in Figure 1), which is regulated by cI itself, and both are expressed in the prophage state. While cI maintains lysogeny, rex offers protection against secondary infection by many phages, originally discovered by the resistance of the lysogen to T4 *r*II phage (hence the name rex, an acronym for ‘*r*II exclusion’) [9,10]. There is some indirect evidence that rex may alter bacterial membrane function [10].

Since both N and cI are important for lysogeny, one would predict that mutation in either gene will abrogate lysogeny. Unexpectedly, however, nearly half a century ago, Lieb and co-workers found that when *E. coli* is infected with λ*N*^-^*c*I^-^ double mutant at a high m.o.i. (multiplicity of infection) of 50 or more phages per cell, almost all the cells survive and apparently stable lysogens appear at a frequency of about 10% [11,12]. Formation of these lysogens require int function and intact attachment sites on the phage genome, suggesting that the prophage genomes are integrated into the bacterial chromosome. These pioneering reports and subsequent studies, including those from our laboratory [12,13,14,15], confirmed that these lysogens indeed contain 20–30 copies of λ*N*^-^*c*I^-^ genomes per cell and were, therefore, named ‘polylysogens’. Several properties of these polylysogens were found to be different from those of nonlysogenic *E. coli* [11,12,13]: (i) these lysogens grow poorly; (ii) they form filaments that are 14–25 times the length of the nonlysogenic parent bacteria; (iii) they produce pinhead-size colonies with an irregular surface; (iv) formation of the polylysogens requires the λ *int* function and the prophage genomes are covalently attached to the bacterial genome; (v) these lysogens occasionally undergo reversion to the nonlysogenic phenotype of faster-growing, normal-sized colonies containing non-filamented cells. These results clearly documented lysogenic “conversion”, defined as phenotypic alteration of the bacterial host as a result of lysogeny. In referring to these lysogens, therefore, we will use the terms “polylysogen”, “converted lysogen”, and “converted polylysogen” synonymously. In this communication, we report detailed studies on the phenotype of these converted lysogens with emphasis on the potential mechanism of conversion. (These studies were part of the Ph.D. thesis research of S.B. [13], carried out under the guidance of N.C.M.).

## 2. Results

### 2.1. Envelope-associated Properties of the Polylysogen

We reasoned that the alteration of the bacterial envelope may underlie the filamentous phenotype and the slower growth of the polylysogens. To test this, we studied the structural and biochemical properties of the polylysogen envelope. In the Gram-negative bacteria, such as *E. coli*, the envelope consists of three distinct layers: the outer membrane, the inner or cytoplasmic membrane, and the peptidoglycan layer that is located between the two membranes and is covalently linked to the lipoprotein of the outer membrane [16]. In this section, we studied several properties of the various layers.

#### 2.1.1. Properties Associated with the Cytoplasmic Membrane

We quantified several transport functions located in the cytoplasmic membrane (inner membrane), which included uptake of small molecules, and the phosphoenolpyruvate (PEP)-dependent phosphotransferase enzyme system (PTS), using the procedures described in Section 4. As its name implies, PTS catalyzes PEP-dependent phosphorylation of sugars along with the coupled transport of the sugar phosphate across the membrane [17]. The PTS consists of three proteins—HPr, enzyme I, and enzyme II—of which the last component is an integral membrane protein [18]. In normal *E. coli*, this enzyme cannot be assayed in fresh intact cells in an in vitro system, but the same can be done with cells that have been subjected to a freeze-thaw cycle. PTS was assayed in polylysogen and control nonlysogenic *E. coli* cells both before and after one freeze-thaw cycle. The results (Table 1) show that the total PTS activity measured in frozen and thawed polylysogens is ~37% of the nonlysogen under identical conditions. Interestingly, ~60% of this total PTS activity could be measured in fresh, saline-washed, non-freeze-thawed cells of the polylysogens, but was essentially undetectable in fresh nonlysogen cells. The polylysogen cells were also about half as efficient as the nonlysogen in the uptake of small molecules (i.e., 60%, 63%, and 52%), respectively for glucose, galactose, and uridine (Table 1). Comparable results were obtained with the activities of specific membrane-bound enzymes; specifically, the activities of four enzymes in the polylysogen membrane ranged from 50%–61% of those in the nonlysogen membrane (Table 1). Together, these results indicate that polylysogeny compromised the integrity of the bacterial inner membrane.

#### 2.1.2. Properties Associated with the Peptidoglycan and the Outer Membrane

The *E. coli* outer membrane is composed of specific proteins, phospholipids, and lipopolysaccharides (LPS) [16,19]. The outer membrane functions as a permeability barrier to a large number of compounds, such as antibiotics, and also provides receptors for bacteriophages. The outer membrane, together with the peptidoglycan, is responsible for the rigidity of the envelope and determines the shape of the bacterial cell. In order to investigate the status of these two layers of the polylysogen envelope, we conducted several experiments, related to the properties mentioned above, namely, autolysis, antibiotic permeability, sensitivity to ionic detergents, and ethylenediaminetetraacetic acid (EDTA). 

**Figure 2:**
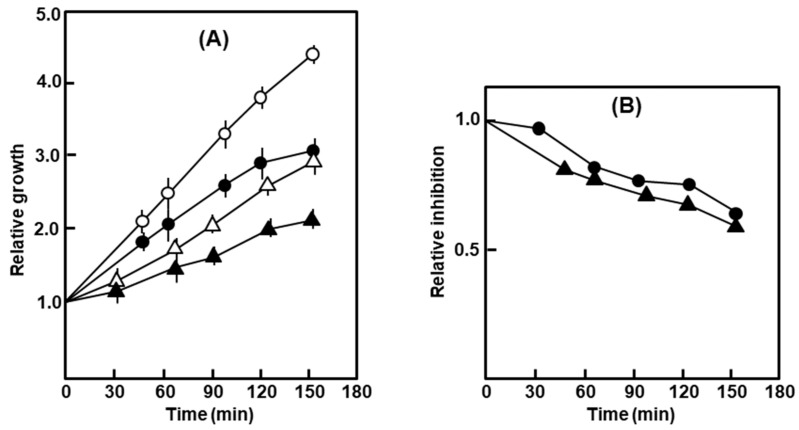
Growth of the polylysogen in the presence of actinomycin-D. (**A**) Relative growth measured by A590 of the culture with time, the starting value (at the time of addition of the antibiotic) being taken as 1. (**B**) Relative inhibition calculated from (A), as the ratio of the average A_590_ of the treated culture at any time and the average A_590_ of the untreated culture at the same time. The symbols are: circles, nonlysogen; triangles, polylysogen; open symbols, untreated; closed symbols, Act-D-treated.

*E. coli* strains are largely impermeable to the antibiotic actinomycin-D, an inhibitor of DNA-dependent RNA synthesis [20]. When added to the growing cultures, the polylysogen and the nonlysogen were both marginally but equally sensitive to the antibiotic (Figure 2). 

**Figure 3:**
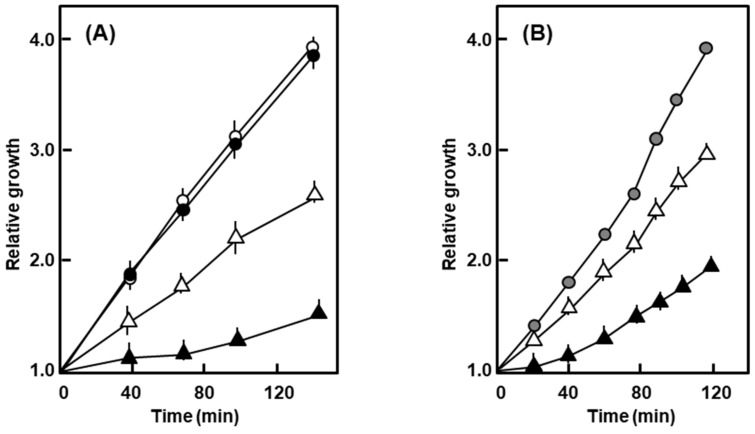
Growth of the polylysogen in the presence of SDS and deoxycholate (DOC). The bacteria were grown in liquid culture in the presence of 0.5% SDS (**A**) and 1% deoxycholate (**B**), and A_590_ followed with time. Symbols: nonlysogen, ο; polylysogen, ∆; empty symbols are for no addition, filled ones are SDS or DOC added. For DOC, the nonlysogen showed a nearly identical growth curve with or without DOC, and therefore, a single graph represents both.

In similar experiments, the growth of the nonlysogen was essentially resistant to 0.5% SDS and 1% deoxycholate (DOC), whereas that of the polylysogen was drastically slowed down (Figure 3). 

**Figure 4:**
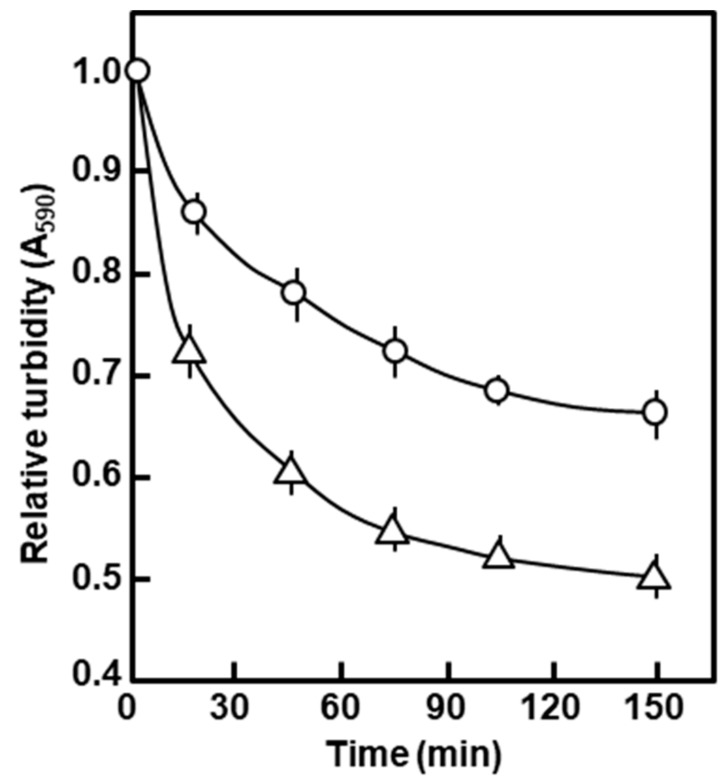
Autolysis of the polylysogen in the stationary suspension. The polylysogen (∆) and nonlysogen (ο) were resuspended and held in phosphate buffer without shaking; turbidity (A_590_) was followed with time, and presented as a fraction of the starting turbidity, taken as 1.

In a standing suspension (i.e., without shaking), 0.5% SDS nearly completely lysed the polylysogen cells in about 5 min, judging by the disappearance of turbidity and increased viscosity (data not shown). Since the increased detergent sensitivity suggested an altered envelope in the polylysogen, we tested the rate of autolysis of these cells. Exponentially growing cells were saline-washed and resuspended in 0.05 M sodium phosphate buffer (pH 7.4); the suspensions were then incubated in a spectrophotometer cuvette at 35 °C, and the change of turbidity (A_590_) followed with time. As shown in Figure 4, the polylysogens autolyzed much faster than the nonlysogen control, particularly noticeable in the initial stages of incubation. The results presented so far point to significant degeneration of the polylysogenic outer membrane.

The space between the outer membrane and the peptidoglycan layer, known as “periplasm”, houses several hydrolytic enzymes, such as alkaline and acid phosphatases, nucleotidases, and cyclic phosphodiesterase [19]. If the integrity of the outer membrane has indeed been affected by polylysogeny, this may be reflected in the relative ease with which the periplasmic enzymes are leaked out of the cell. The polylysogen and control nonlysogen were, therefore, treated with 0.5 M EDTA, and the periplasmic enzymes were assayed in the supernatant, to test if they were in fact released. Results (Table 2) show that the polylysogen releases a larger fraction of its periplasmic enzymes, whereas the nonlysogen releases little or none. The cytoplasmic enzymes were either not released (e.g., G6PD, glucose-6-phosphate dehydrogenase) or equally released (e.g., inorganic pyrophosphatase) by both the cells. 

As mentioned earlier, the *E. coli* outer membrane also contains phage receptors that are composed of LPS, proteins, and lipoproteins. When we tested the plating efficiency of phage T4D, the polylysogens plated more poorly than the monolysogen (containing a single copy of wild type lambda prophage (Table 3); the two T-odd phages, namely T3 and T7, however, plated with equal efficiency.

**Figure 5:**
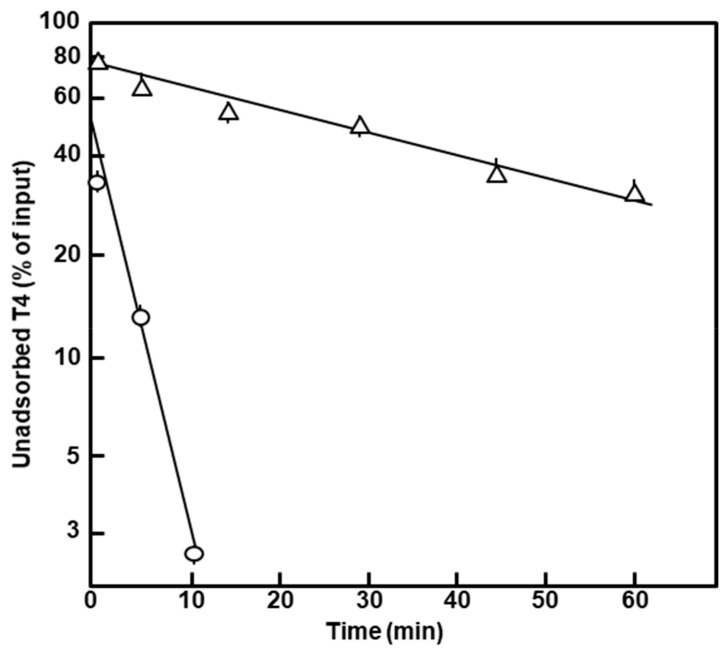
Kinetics of T4 phage adsorption to intact cells. T4D phage was added to the polylysogen (∆) and nonlysogen (ο) exponential phase cultures at an input m.o.i. (multiplicity of infection) of approximately 0.5, and incubation continued without shaking. Aliquots were taken out at the indicated times, chilled and immediately centrifuged at 10,000× *g*, for 2 min in a prechilled microcentrifuge. The unadsorbed phage in the supernatants was assayed for pfu by the usual methods, and the calculated concentration as the percent of the input was plotted. Note that it took approximately 1 min to withdraw a sample and start centrifugation, and thus, the earliest data point is for 1 min, not 0 min.

The lower plating efficiency of T4 was reflected in the slower kinetics of adsorption of the phage (Figure 5). The T4 plating and adsorption results suggest changes in the phage receptor, further supporting an alteration of the polylysogen outer membrane. 

#### 2.1.3. Envelope Composition

In an attempt to determine if the functional changes in the polylysogen envelope were reflected in its composition, we first studied the LPS. Results (Table 4) show that the purified envelope from the nonlysogen contained 5% of its dry weight as LPS, while in the polylysogen, the LPS constituted 3.5% of the envelope dry weight, which is ~72% of the nonlysogen value. We also found that equal amounts of the two LPS samples contained nearly equal amounts of total carbohydrate (~32% of dry weight). They also showed essentially identical composition in terms of monosaccharides, although two sugars, namely galactose and colitose, could not be detected for unknown reasons. 

**Figure 6:**
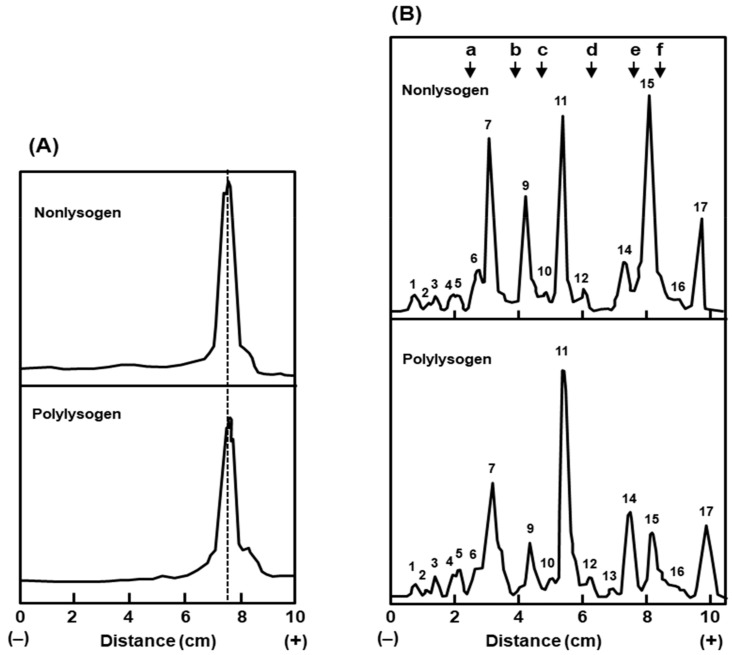
The LPS **(A)** and outer membrane proteins **(B)** of the polylysogen and nonlysogen were analyzed by gel electrophoresis as described in Section 4.2.3. The stained bands in the respective gels were densitometrically scanned and plotted as shown. Protein molecular weight markers (indicated in the top right panel) and their sizes were as follows: (a) bovine serum albumin, 68k; (b) ovalbumin, 45k; (c) pepsin, 34.7k; (d) trypsinogen, 24k; (e) β-lactoglobulin, 18k; (f) lysozyme, 14.3k. The major protein bands were serially numbered (1–17) by their migration distance from the cathode.

As the carbohydrate compositions of LPS in the two bacteria were quantitatively similar, we explored whether they have qualitative differences. The two major structural units of the LPS are the oligosaccharide core and lipid A [21]. An alteration in the chain length of either of these should affect the size of the total LPS. When we compared the relative mobility of the purified LPS preparations of the two bacteria by the SDS-PAGE procedure [22], they were essentially indistinguishable by size (Figure 6), which also corroborated with the similar carbohydrate composition of the two. It is, therefore, unlikely that the polylysogenic conversion is related to a change in the LPS.

Lastly, we attempted to identify the differences in the proteins of the envelope. It should be noted that in contrast to the inner membrane, which contains a large number of proteins in small amounts, the outer membrane consists of fewer protein species in large amounts [23]. Thus, in SDS-PAGE analysis of the total envelope, the outer membrane protein bands are easily observed above the baseline of inner membrane proteins, making their detection easier [23,24]. Our results (Figure 6), reveal that the outer membrane protein patterns of the two bacteria are significantly similar, as judged by their relative mobility. However, upon densitometric analysis of the peaks, several major bands showed quantitative differences (Table 5), which included higher amounts of peaks 11 and 14, and lower amounts of peaks 9 and 15, all in the polylysogens, compared to the nonlysogen.

We conclude that the appreciable differences between the outer envelope of the nonlysogen and the polylysogen are in the amounts of specific outer membrane proteins, and not in the LPS.

### 2.2. Characteristics of the Polylysogen Filaments

#### 2.2.1. Filament Length

**Figure 7:**
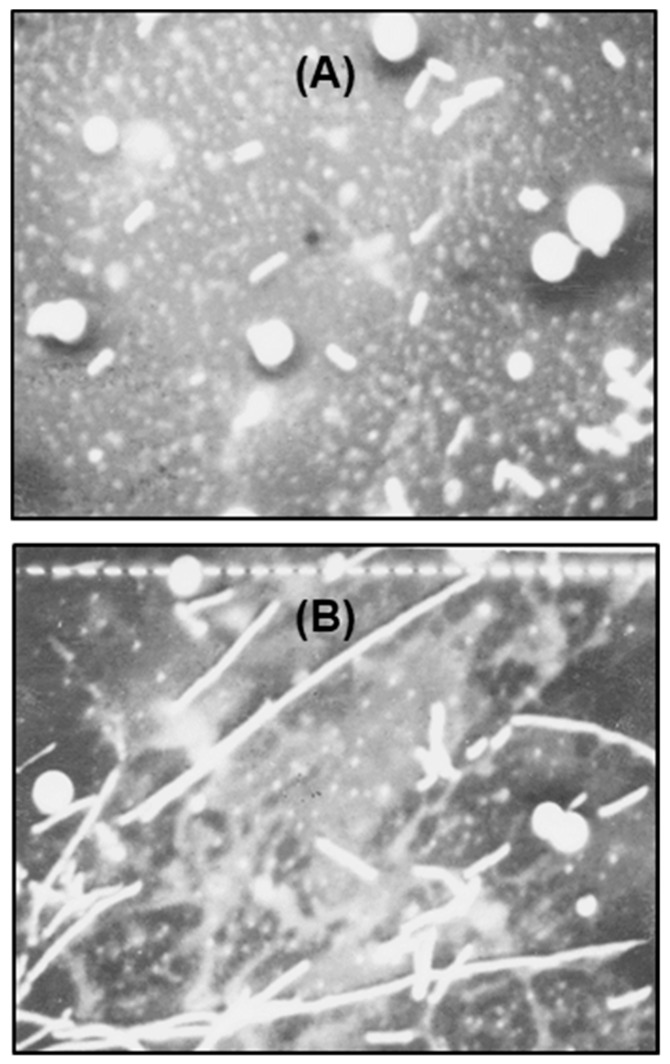
Scanning electron micrograph of the (**A**) nonlysogen and (**B**) polylysogen cells, prepared as described in the Section 4.2.2. Total magnification = 1600×; one small bar (B) = 1 μm. The spheres are artifacts of sample preparation and should be ignored.

The polylysogenic filament has not been studied in depth, although filamentation indicates a defect in cell division. Proper division of progeny bacterial cells is orchestrated by multiple biochemical events, functioning in a well-timed and spatially organized scheme; the major steps of which are: DNA replication and septation before the separation of the two daughter cells, followed by physical detachment of the two cells [25]. In this section, we investigate some of the properties of the filamentous nature of the polylysogen, in an effort to understand the defect in cell division. We first measured the length of the filaments by multiple approaches, at first by scanning electron microscopy. As shown in representative micrographs (Figure 7), both nonlysogen and polylysogen cells were similar in diameter (0.8 μm); however, whereas the former cells had a relatively uniform length of 2.7 μm, the filaments of the latter ranged in length from 37 to 67 μm (i.e., approximately 14–25 times longer than the nonlysogen cells).

We also noticed early on that a polylysogen culture is always a mixture of filaments of various lengths, which became shorter with continuous culture. As we will show later, this is due to the gradual and spontaneous loss of the prophage genomes, a process we have termed ‘deconversion’ (Section 2.3). In a complementary approach, we measured the colony-forming units of two bacterial cultures by plating serial dilutions on agar plates. In this particular test, the nonlysogen culture produced 5.5 × 10^8^ colonies per A_590_ unit, whereas that number for the polylysogen culture was 2.8 × 10^7^, about 20 times lower. This is consistent with the greater lengths of the polylysogen cells, averaging ~20 cell length per filament, each such filament forming one colony. Parenthetically, these results also suggested that all the filaments in a polylysogen culture are viable. We would like to add that in plating the cells from a polylysogen culture, ~70% of the colonies were visible and could be easily counted after 16–20 h, but more colonies showed up on continued incubation, and essentially all appeared by 36 h. Thus, for full colony count, we incubated the plates for ~36 h so that even the tiniest colonies became visible. As implied above, at any given point of incubation, the colonies that originated from a polylysogen culture were of diverse sizes, from very tiny to nearly the size of a nonlysogenic *E. coli* colony, when grown for the same period of time. It may be mentioned that the shortening of filament length is also due to continuous deconversion of the converted polylysogens, as presented later (Section 2.3).

#### 2.2.2. Irregular Placement and Uniqueness of Septa in the Polylysogen Filament

**Figure 8:**
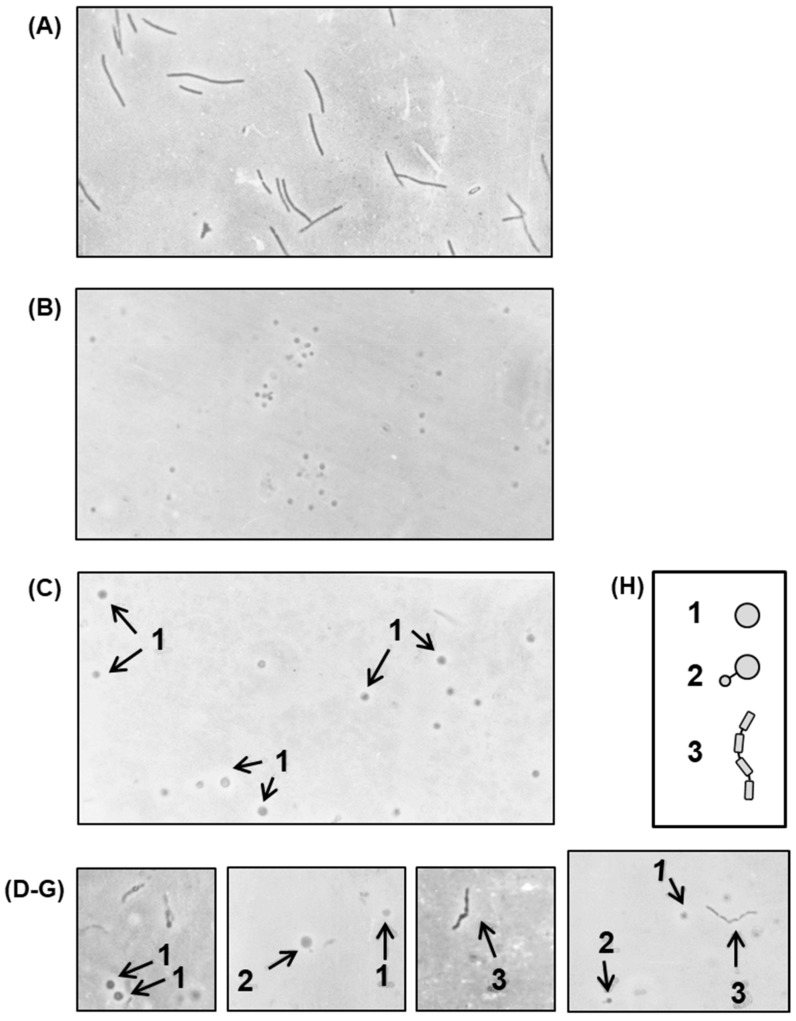
Cells were grown in the presence of nalidixic acid (NA), and then treated with lysozyme-EDTA to convert into spheroplasts, as described above. The photomicrographs represent (**A**) NA-induced nonlysogen (594) filaments, befor*e* spheroplasting. All other micrographs show spheroplasted cells from the following: (**B**) nonlysogen, not exposed to NA; (**C**) NA-induced nonlysogen filaments; (**D**–**G**) polylysogen, not exposed to NA. The various shapes, generated by spheroplasting are sketched for ease of recognition (**H**) and marked by numbers, as follows: 1, giant spheroplast; 2, asymmetric dumb-bell shape; 3, beaded chain. Magnification = 475×.

The septum forms the dividing wall between two daughter cells of a bacterium, which must be split to separate the cells in the final step of division. To identify the nature of the defect in cell division that led to filamentation, it was clearly important to understand the status of the septa in the filament of the growing polylysogen. We first wanted to know whether the filaments were fully septated (i.e., every normal septal site was engaged in the process), or only partially septated, or perhaps not septated at all. Treatment of Gram-negative bacteria with lysozyme-EDTA under controlled conditions [26] led to the loss of the cell wall, and hence, the rod shape of the cells was lost and converted into a spherical shape, known as a ‘spheroplast’. If a filament is devoid of septa, lysozyme-EDTA treatment will lead to a giant spheroplast, whereas if it is septated to any extent, a beaded chain will form, and this will lead ultimately to the formation of spheroplastic bulges at the septal regions. To have an authentic non-septated filament as a control, *E. coli* 594 was grown in the presence of nalidixic acid (abbreviated as NA; 20 μg/mL) for 3 h. Photomicrographs of the spheroplasts (Figure 8) show that the nonlysogen under nontreated and NA-treated conditions gave rise to small and giant spheroplasts, respectively, as expected. 

Thus, the spheroplasts from the polylysogen, in contrast to those from the nonlysogen, present a variety of structures (e.g., giant spheroplasts, dumb-bell shapes, as well as beaded filaments). These structures were likely derived from filaments that were completely nonseptated (giant spheroplasts), partially septated (dumb-bell shapes), and fully septated (beaded filaments). The septation process in the polylysogen, therefore, appears to be irregular or haphazard. 

Filaments of diverse bacterial species, resulting from mutations or nutritional shifts, have been shown to resume division after the addition of various small molecule effectors [25], such as NaCl [27], methanol and ethanol [28], pantoyl lactone [29], dimethyl sulfoxide [28], sodium dodecyl sulfate [30], spermine, and spermidine [25]. We tested all of these chemicals at the concentrations recommended in the publications, but none could promote division of the polylysogenic filaments (data not shown), underscoring a structural distinctiveness in septal adhesion.

#### 2.2.3. Division Status in Different Segments of the Polylysogen Filament

**Figure 9:**
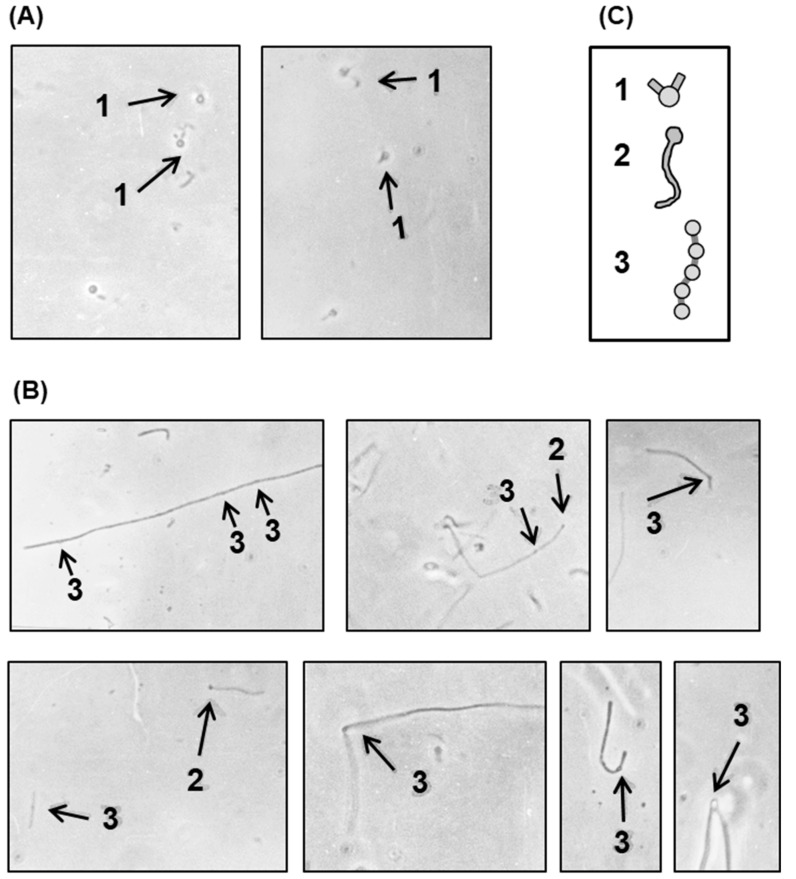
Bacteria were grown in the presence of penicillin as described above, and photomicrographs of nonlysogen (**A**) and polylysogen (**B**) cells were taken. The various shapes are sketched for ease of recognition (**C**) and marked by numbers, as follows: 1, rabbit ear shape terminal bulge; 2, terminal bulge; 3, internal bulge. Magnification = 475×.

In spite of the previous demonstration (Section 2.2.1.) that all the filaments in a polylysogen culture are viable and that many of them were septated, we did not know whether there were preferred sites of division in the filaments. For example, they might be dividing only at the termini or only in the central regions. To resolve this, we capitalized on the fact that penicillin produces spheroplasts from sensitive cells growing in isotonic media, and therefore, the positions of spheroplast bulges would indicate the envelope growth sites. In this experiment, we grew the polylysogen (and the control nonlysogen) in tryptone broth [31] containing NaCl at a final concentration of 0.15 M. Following the addition of penicillin (180 μg/mL), the nonlysogen culture was grown for 2 h, and the polylysogen, for 3 h (because of its slower growth rate), after which the cells were photographed. Results (Figure 9) show that the nonlysogen cells uniformly produced the typical ‘rabbit-ear’ shapes with bulges in the middle of the rods. The polylysogen, on the other hand, showed bulges at various positions in different filaments, which were terminal as well as internal. We conclude that the division of the polylysogen filamentous cell occurs from any of the several sites in a random fashion. 

### 2.3. Deconversion of the Polylysogen

As alluded to earlier, when a typical culture of the polylysogen was spread onto agar plates after proper dilution, and the plates were incubated for about 36 h, colonies of heterogenous sizes could be seen. On microscopic examination, it was found that the smaller the colony, the longer the filaments in it; conversely, the larger colonies had more of the shorter filaments. The few very large colonies contained mostly small rods, typical of the parent nonlysogen, *E. coli* 594. These results indicate that the converted polylysogenic state is metastable and spontaneously degenerates with growth or subculture. This phenomenon has been termed as ‘deconversion’, and the resulting bacteria, ‘deconverted’ bacteria. In anticipation that understanding the deconversion process might shed light on the mechanism of conversion itself, we performed a series of detailed studies of deconversion. To this end, bacteria of three stages of deconversion were selected as follows. The maximally converted culture was obtained by picking a large number of the smallest colonies from the plate, inoculated in liquid medium, and grown for 6-8 h during the day. A parallel culture, subcultured and grown for longer periods, produced bigger colonies on the plate, some the size of regular *E. coli* 594 colonies. These colonies were picked and purified by the usual technique of single colony isolation for a few days, until no small colonies appeared. This population was designated ‘fully deconverted’ or simply ‘deconverted’ (abbreviated as D). Those at an intermediate stage were collected as ‘semi-deconverted’ (abbreviated as SD). Note that due to the continuously degenerating nature of the converted phenotype, these populations overlapped to various extents and therefore, were somewhat variable between experiments. Nevertheless, they offered us three gradual stages of deconversion, amenable to studies at the population level, and some of their properties are listed in Table 6.

**Figure 10:**
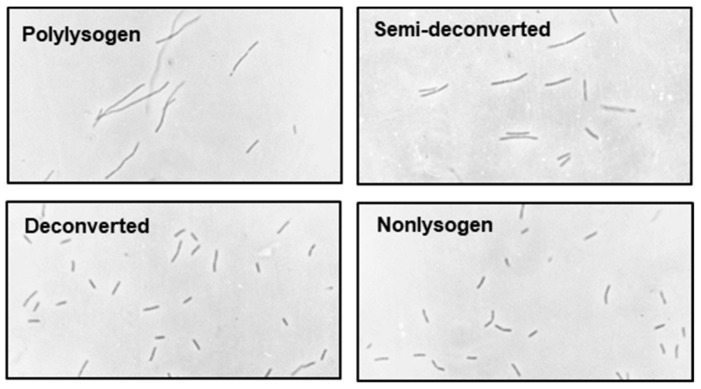
Photomicrographs of polylysogen at different stages of gradual deconversion, as labeled. Note that the fully deconverted cells are essentially indistinguishable from those of the nonlysogen.

We first focused on the most visible converted phenotype, namely, small colonies. By virtue of their very selection, the polylysogen, polylysogen-D, polylysogen-SD, and nonlysogen 594 cultures were expected to produce increasingly larger average size of colonies, which was confirmed. Concomitantly, the length of the filament also decreased, and the polylysogen-D cells regained the small rod shape, indistinguishable from the nonlysogen; the cell lengths were measured from the photomicrographs (Figure 10) and presented in Table 6. 

Upon quantification of the prophage genome copies by hybridization with purified λDNA (as described in Section 4), the number was found to decrease as deconversion progressed, being undetectable in polylysogen-D (Table 6). The efficiency of plating (E.O.P.) of plating of T4 and leakage of alkaline phosphatase upon treatment with 0.5 M EDTA (Section 2.1.2.) also followed the same trend (Table 6). We conclude that the full gamut of the polylysogenic phenotypes is dependent on the presence of the large number of the λ*N*^-^*c*I^-^ prophage genomes.

### 2.4. Role of Lambda Genes in Conversion

As presented earlier (Section 1 and Figure 1), the most likely λ gene products to be expressed from the λ*N*^-^*c*I^-^ prophage genome are: rex, from the *prm* promoter that it shares with cI; cro, transcribed from the pR promoter, which does not require N function because it is located upstream of the *tR*1 terminator; and finally, small amounts of terminator-downstream genes that may be expressed by inefficient termination (~10% of transcription initiated from *p*L, and ~40% of that from *p*R), as mentioned earlier. 

We started with interrogating the role of rex, and it was unlikely that a significant amount of rex would be produced, for two reasons [2,4]: (i) in the lysogenic state, an active cI repressor is needed to turn off rightward transcription from the *P*_R_ promoter with simultaneous channeling of the leftward transcription from *prm* promoter, which transcribes the *rex* and *c*I genes. In this polylysogen, the *c*I gene is nonfunctional. Under this condition, to test the possibility that rex may have any role in the conversion process, the functional level of rex protein in this polylysogen was determined by measuring the E.O.P. of T4*r*II phage. The results showed that while the E.O.P. of this phage on 594(λ^+^), was 7.7 × 10^−9^, on the polylysogen it was 1.3 × 10^−2^. This indicates that the rex function is much weaker in the polylysogen, compared to the wild type lysogen, 594(λ^+^). Considering that the polylysogen binds T4 phage only about a fifth as efficiently as the nonlysogen (Table 6), the higher efficiency of T4*r*II plating is even more remarkable, further underscoring a weaker expression of rex. Hence, the converted phenotypes of the polylysogen cannot be attributed to rex function.

**Figure 11:**
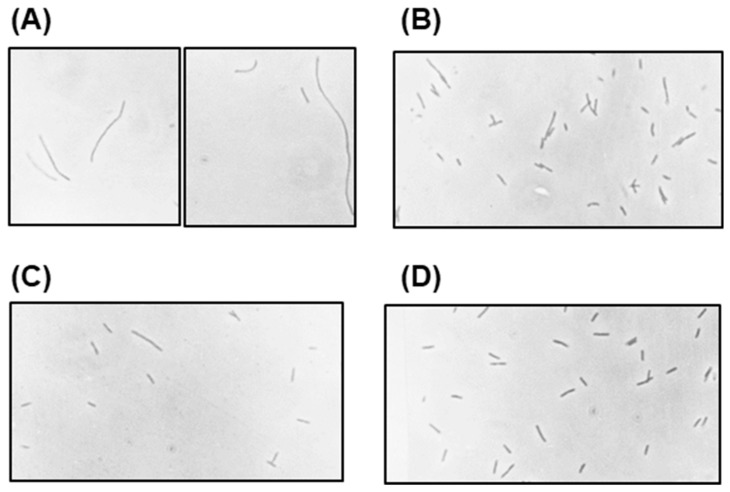
Photomicrographs of different polylysogens, compared to the nonlysogen. Bacteria from freshly grown cultures of the following were photographed at 475×: (**A**) indistinguishable cells of 594(λ*N*^-^*c*I^-^) or 594(λ*N*^-^*c*I^-^*R*^-^) or 594(λ*N*^-^*c*I^-^*S*^-^) or 594(λ*N*^-^*c*I^-^*R*^-^*S*^-^), presented in a single panel representing all, to save space; (**B**) 594(λ*N*^-^*c*I^-^*O*^-^); (**C**) 594(λ*N*^-^*c*I^-^*P*^-^); (**D**) 594.

Next, we proceeded to test if any of the *O*, *P*, *R*, and *S* genes have any role in conversion. Studies in our laboratory showed that in the polylysogen, both phage-specific and host-specific DNA replication machineries are active and operate independently of each other; furthermore, the O and P functions are also needed for the maintenance of the full complement of 20–30 copies of prophage genomes [14]. The *R* and *S* gene products cause cell lysis [32], and thus, it is possible that their suboptimal expression from the multiple *N*^-^ genomes may cause the observed alterations of the polylysogen cell wall [33]. To complement our data on *O* and *P* genes [14], and to test the possible role of R and S, we tested the biochemical phenotypes of conversion in lysogens of the configuration 594(λ*N*^-^*c*I^-^*X*^-^), where *X* is *O*, *P*, *R*, or *S*. The morphologies of the cells (Figure 11) show that loss of either R or S or both had no effect on the length of the filaments (i.e., they were essentially of the same length as the *N*^-^*c*I^-^ polylysogen), whereas the loss of O or P led to shortening of the polylysogen filaments. 

As in deconversion, the size of the colonies paralleled the copy number (i.e., mutations in R or S created small colonies typical of the parent polylysogen), whereas those with mutations in O or P led to larger colonies, close to the size of nonlysogenic 594 colonies (Table 7). Other conversion-associated properties of these mutants are summarized in Table 7.

Results (Table 7) reveal that R and S had little or no role in conversion since the polylysogens containing prophages that were mutated in these genes were essentially indistinguishable from the original 594(λ*N*^-^*c*I^-^). Mutations in either of the two replication-related genes, *O* and *P*, appeared to have affected lysogeny, since mutations in either of them led to lysogens with a lower number of prophage genomes; the properties of these ‘oligolysogens’ were somewhere in between a fully converted 594(λ*N*^-^*c*I^-^) polylysogen and the nonlysogen, 594. The *O* and *P* genes were previously shown to be needed for maintenance of the multiple prophage genomes in the converted polylysogen [14]. It is thus obvious that in the absence of O and/or P, the phage-mediated (active) replication of the lambda prophage genomes will stop, and therefore, the copy number of prophages in absence of O and/or P will be reduced, since they will be maintained by passive replication only, activated by the host replication machinery. In fact, in the absence of either O or P function, the oligolysogens that are formed have been shown to contain only 5–6 copies of integrated prophage genomes per cell [14]. In this scenario, we could not conclude whether the partial loss of conversion is a direct effect of loss of the *O* and/or *P* gene products or an indirect effect of lower copy number of the prophages, which could have reduced the level of another lambda gene product due to the lower gene dosage. 

## 3. Summary and Discussion

**Figure 12:**
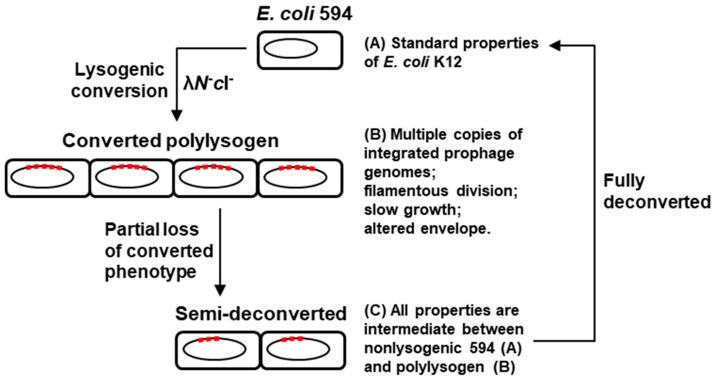
Schematic model of polylysogenic conversion and spontaneous deconversion, summarizing the key results reported in this paper.

In this paper, we presented a series of studies on the properties of an unusual polylysogen of phage lambda, namely 594(λ*N*^-^*c*I^-^), and the main findings are as follows: (a) The converted phenotypes include filamentous growth, small colonies, high copy number of the integrated prophage, and alteration of both inner and outer membranes of the cell well; (b) The conversion is metastable in that the polylysogens undergo apparently spontaneous ‘deconversion’, in which they progressively lose both the prophages and all the converted phenotypes; (c) Among the few genes that may be expressed in the *N*^-^*c*I^-^ prophage genomes, only *O* and *P* were important for maintenance of the high copy number of the prophage, and thus, it could not be concluded whether they have a direct role in bringing about the converted properties; (d) The *rex* gene and the lysis genes, *R* and *S*, had no role in conversion. Some of the major findings are summarized in the schematic diagram (Figure 12).

The polylysogenic conversion differs from known examples of conversion in several respects. As a general rule, very few genes are expressed from prophage genomes, most of which, like that of phage λ, replicate as part of the chromosome of their host bacteria [34]. In principle, lysogenic conversion can be caused either directly by the phage gene products that are expressed in the prophage, or more rarely, by host functions that are induced or activated by the prophage. In most cases, one or two host properties undergo changes, usually providing a net benefit to the cell. Thus, lysogenization by wild type λ can be viewed as conversion that endows *E. coli* with immunity against superinfection by homologous phages as well as the capacity to exclude *r*II mutants of T4 phage [10]. No other properties of *E. coli* have been reported to be altered in a λ^+^ lysogen. In several other cases, prophage gene products regulate bacterial virulence, biofilm formation, and evolutionary and reproductive fitness [34,35,36,37,38]. The classic examples may be cited as follows. The epsilon (ε) phages of *Salmonella* code for enzymes that alter the O-antigen sugar chains and the cell wall, which alters the host range [39]. Severe pathogenicity of other bacteria [40] such as *Corynebacterium diphtheriae* [41], *Vibrio cholerae* [42], and *E. coli* O157:H7 [43] is due to the presence of the specific prophages encoding toxin genes. In contrast, conversion in the polylysogen is much more extensive, since a large number of host properties appears to have altered, although we do not know if the multiple alterations are actually the pleiotropic effects of changes in one or two key host molecules or enzymes. Furthermore, the altered properties do not appear to offer any significant benefit to the host, as the polylysogen grows poorly, has a more fragile cell wall, is less sensitive to T4 but more sensitive to T4*r*II. Lastly, the polylysogen is a rare example where the prophages in the derepressed (*c*I^-^) condition remain in the integrated state. However, their active replication (by phage *O* and *P* genes) and maintenance thereof are finely regulated by Cro in the absence of cI [14].

The reception of bacteriophages T4, T3, and T7 all require interaction with the basal core of the LPS. Since the LPS of the polylysogen and the nonlysogen were indistinguishable in both quantity (Figure 6) and composition (Table 4), and since the plating efficiencies of T3 and T7 were also similar in the two bacteria (Table 3), the differential T4 plating efficiency seems to be very specific for the T4 phage and perhaps not directly related to LPS. In contrast to LPS from *E. coli* strain B/r, that from strain K12 was shown to require a major outer membrane protein, namely protein Ib (also known as O8), for T4 reception [44,45]. This protein is the same as our protein peak number 9 (Figure 6), with a Mr of 39,000, which also corresponds to peak 4 of Inouye [23]. It is possible that the relatively lower amount of this protein in the polylysogen (Table 5) underlies the lower efficiency of T4 plating, but additional experiments are needed to establish this fully.

Our studies on several lambda genes to understand their role in conversion did not identify any such gene but ruled out a number of possibilities. Here, we would like to assess the possible role of a few other candidate genes in polylysogenic conversion. The role of the λ*cro* gene could not be directly verified because λ*c*I^-^*cro*^-^ phage is non-viable. However, cro is most probably not involved because a mutant λ*N*^-^*c*I^-^ which also contained the constitutive mutation *v*1*v*3 in the right operator (that does not bind the cI repressor), maintains a much higher cro level, but did not convert [14]. Two genes in the early left operon, *kil* and *hin*, which reside downstream of the *t*L termination site, and therefore, may produce only small amounts of mRNA in the absence of N, deserve mention due to their known interactions with *E. coli* physiology. The *kil* gene was shown to be necessary for host killing under *N*^+^*O*^-^ or *N*^+^*P*^-^ conditions [46]. Interestingly, *kil* gene product also causes filamentation of *E. coli* [47]. However, this filamentation is quite different from that of the converted lysogen as pantoyl lactone could induce division in the former but not in the latter (Section 2.2.2). The *hin* gene product was shown to inhibit the transport of amino acids and nucleotides into the cell [48], believed to be caused by an alteration of the cytoplasmic membrane. The possibility that *kil* or *hin* gene products are expressed in appreciable amounts in the converted polylysogen has not been tested. In addition, it could be tested whether combining *N*^-^*c*I^-^ with *kil*^-^ or *hin*^-^ mutations abrogates conversion. Since the lysis-related *R* and *S* gene products, by the very nature of their function, act upon the cell envelope, they received our initial attention; however, they were found to have no effect, as their mutation did not affect polylysogenic conversion. It is known that the S protein can damage the cytoplasmic membrane, allowing the R protein, the phage-coded lysozyme, to exit into the periplasmic space to act on the peptidoglycan layer, thereby effecting cell lysis [33], but this is dependent on functional N protein. Thus, our results are probably not unexpected, since the expression of these two genes (*R* and *S*) in the absence of the N antiterminator (i.e., in the *N*^-^*c*I^-^ polylysogen) would be an impossible event as rightward transcription is completely terminated at the *tR*2 termination site under this condition.

In preliminary studies that are not reported here, we also obtained evidence for the potential role of host gene(s) in conversion. Specifically, when we tested various K12 strains of *E. coli,* 594 and W3350 could be readily converted by infection with λ*N*^-^*c*I^-^. When the genotypes of all strains were examined, these two strains were found to contain *gal*K^-^ and *gal*T^-^ mutations. These two genes of the *gal* operon respectively code for galactokinase and galactose-P-UDP transferase and are essential for galactose catabolism. When we changed 594 (*gal*K^-^ *gal*T^-^) to 594 (*gal*^+^) by transduction of the wild type genes, the resultant strain could no longer be converted by λ*N*^-^*c*I^-^, although we did not test the prophage status. How the absence of functional galactokinase and galactose-P-UDP transferase enzymes may help in the conversion process would be an exciting study. There is a possibility that the phage gene-mediated replication of integrated lambda genomes may continue into the arrays of genes (*gal* and *bio*) that bracket the prophages and may cause replicational escape expression of those host genes, one or more of which may have specific role(s) in conversion. It is also possible that in the absence of *gal*K and *gal*T, even a mild expression of *gal*E through replicational escape may cause conversion.

Although the mechanism of the gradual loss of prophage genomes, as observed in our studies, remains unknown, this could be due to intra-prophage recombination, causing removal of one or more of the prophage genomes during continued growth. This is supported by the fact that in these polylysogen cells, ~10% of the total prophage genomes always exists as plasmid, the size of which is about 4–5 phage genome length [12].

In conclusion, our findings have left the door open for the exact molecular mechanism of polylysogenic conversion; specifically, questions to be addressed in future studies include: (a) Which lambda gene or genes, if any, are involved in the conversion? (b) Are cellular (*E. coli*) genes also involved in the process? (c) How do these products cause the alterations of the cell envelope? (d) Why does the polylysogen lose the prophage genomes? Further studies aimed at answering these questions will certainly unravel novel mechanisms of interaction between *E. coli* and bacteriophage lambda, the classic host–virus duo that paved the way to modern molecular biology, related to gene regulation [1].

## 4. Materials and Methods

### 4.1. Chemicals, Bacteria, and Phages

The following chemicals were from Sigma (St. Louis, MO, USA): D-(+)-galactose, D-(−)-lactate (Li salt), maltose, 2-deoxy-D-glucose, Tris, PMS (phenazine methosulfate), MTT [3-(4,5-dimethylthiazol-2-yl)-2,5-diphenyltetrazolium bromide), β-mercaptoethanol, TEMED (tetramethylethylenediamine), sodium deoxycholate, actinomycin-D, NADH, ATP, cAMP, DMSO, pantoyl lactone, spermidine, pNPP (para-nitrophenylphosphate), lysozyme, and Dowex-1-chloride. The other chemicals were purchased from the following manufacturers: D-glucose, sucrose, EDTA, ascorbic acid (BDH, Glaxo laboratories India Ltd., Mumbai, India); sodium arsenite, sodium molybdate, ammonium persulfate (E. Merck, Darmstadt, Germany); bacterial and phage growth media components (Difco Laboratories, Detroit, MI, USA); acrylamide, bis-acrylamide (Eastman Kodak, Rochester, NY, USA); Coomassie Brilliant Blue (Colab, Chicago Heights, IL, USA); Eosin Y (Allied Chemicals, Morisstown, NJ, USA); methylene blue, SDS (Centron Research Laboratories, Mumbai, India); all radiochemicals (Bhava Atomic Research Center, Trombay, Mumbai, India). 2-Keto-3-deoxy-octonate (KDO) was kindly provided by Dr. Anadi N. Chatterjee, Department of Microbiology of our Institute.

Various bacteria and phages were generous gifts from various investigators as follows: *E. coli* 594 (*Su*^-^), C600 (*Su*II^+^), Ymel (*Su*III^+^), λ^+^ and λ*Nsus*7*Nsus*53*c*I60 (abbreviated here as λ*N*^-^*c*I^-^) were from Dr. Margaret Lieb (University of Southern California School Medicine, Los Angeles, CA, USA); suppressor-sensitive *Rsus*5 and *Ssus*7 (abbreviated here as *R*^-^ and *S*^-^) alleles, T4D, and T4*r*II were from Dr. Sankar Adhya (NIH, Bethesda, MD, USA); T3, T7 from Dr. Umadas Maitra (Albert Einstein College of Medicine, Yeshiva University, Bronx, NY, USA). 

### 4.2. Methods

#### 4.2.1. General and Common Procedures

Unless otherwise mentioned, all routine bacteria and phage procedures were performed as described [13,14,15,31], and were also described in detail (PhD thesis). Protein concentrations were measured according to Lowry [49]. Assays were done three times, and the average is presented with standard error; some numbers were rounded for the sake of brevity. The various suppressor-sensitive λ*sus* mutants (listed in Section 4.1.) were grown in the appropriate suppressor *E. coli* strain (C600 *Su*II^+^ or Ymel *Su*III^+^). Efficiency of plating (E.O.P.) of a phage was determined by normalizing the number of plaques to the same dilution and then using the relation: E.O.P. = (pfu on the experimental bacteria, e.g., the polylysogen)/(pfu on control bacteria, e.g., 594). In all the graphs, each point represents the average of three experiments, with standard error bars as shown. Results with *p*-value < 0.02 were considered significant.

#### 4.2.2. Visualization and Microscopy of Bacteria

Bacteria in liquid culture were counted with the help of a hemocytometer grid under a microscope. For light microscopy, the culture was spread on a standard slide, covered with a coverslip, and photographed in a Leitz Ortholux phase-contrast microscope. For scanning electron microscopy, bacteria were fixed with 2% glutaraldehyde for 1 h at 4 °C, resuspended in saline at about 10^6^ cells /mL; 2 μL of the suspension were spread on the electron microscopic slide and held at room temperature for 3 days. The slide was processed by standard gold-coating procedure and scanned in a Philips scanning electron microscope Model PSEM 500.

#### 4.2.3. Isolation and Analysis of Bacterial Envelope and Its Constituents

The published procedure [26] was followed with minor modifications as follows. The spheroplasts, produced upon lysozyme-EDTA treatment, were lysed by pouring into four volumes of ice-cold water. The lysed suspension was centrifuged at 1200× *g* for 15 min, and MgCl_2_ (5 mM final) were added to the supernatant, which was then centrifuged at 110,000× *g* for 40 min. The membrane pellet was suspended in 0.02 M Tris, 1 mM EDTA (pH 8), and the addition of MgCl_2_ and centrifugation was done as in the preceding step. The final pellet was resuspended in 10 mM Tris-Cl, pH 8, and the suspension used for the assay of membrane-bound enzymes. 

The LPS was purified from bacterial membranes by the phenol-chloroform-petroleum ether (PCP) extraction method, as described previously [50]. LPS was analyzed by SDS-PAGE and the bands were visualized by periodate-Schiff stain [22]. The carbohydrate constituents of the LPS were quantified using the published procedures, as referenced here: total carbohydrate [51]; rhamnose [52]; heptose [53]; glucose, using glucose oxidase [54]; galactose, as in glucose, but using galactose oxidase instead [55]; colitose [55]; KDO [56].

Among the periplasmic enzymes, alkaline phosphatase was assayed by the method of Garen and Levinthal [57], and 5′-nucleotidase, cyclic phosphodiesterase, and acid phosphatase were assayed according to Neu and Heppel [58]. Among the cytoplasmic enzymes, inorganic pyrophosphatase was assayed in a total reaction volume of 1 mL, which contained 100 μmoles Tris-Cl (pH 7.5), 2 μmoles inorganic phosphate and an appropriate amount of membrane preparation. The reaction was incubated at 37 °C for 10 min, and then stopped by adding 1 mL 0.4 M trichloroacetic acid (TCA). The rest of the procedure, including the estimation of inorganic phosphate in the presence of (remaining) pyrophosphate, was exactly as described above for the assay of 5′nucleotidase. Glucose-6P-dehydrogenase (G6PD) was assayed as described [59]. Among the membrane-bound enzymes, D-lactate dehydrogenase (LDH) was assayed by the method of Kohn and Kaback [60], modified as follows. The reaction mixture (1.2 mL) contained 12 μmoles D(-) lactate (Li-salt), 100 μmoles potassium phosphate (pH 7.8), 20 μg FAD (flavin adenine dinucleotide, di-sodium salt), 70 μg 1-methoxy phenazine methosulfate (PMS), 40 μg 3-(4,5-dimethylthiazol-2-yl)-2,5-diphenyltetrazolium bromide (MTT), and 50 μL of membrane preparation. The mixture was incubated at 37 °C and the increase in absorbance at 550 nM followed with time. In the blank, lactate was replaced by water. NADH (nicotine adenine dinucleotide, reduced) oxidase was assayed according to Gutman et al. [61], and both dye (tetrazolium chloride) reductase and ATPase were assayed according to Datta and Franklin [62]. To measure the PTS activity, exponential phase bacterial cells were washed and resuspended in normal saline, and a portion was used to measure the accessible PTS activity in fresh cells, while total PTS activity was measured in the other portion that was frozen and thawed. The assay procedure was as described by Ghosh and Ghosh [63]. 

The absence of cytoplasmic, inner membrane, and periplasmic material in the purified outer membrane fraction was tested and confirmed by assaying for the respective marker enzymes (i.e., LDH, G6PD, and alkaline phosphatase), which were undetectable (<2% of the level in their cognate fractions) (data not shown). Likewise, the inner membrane fraction was also free of periplasmic alkaline phosphatase.

#### 4.2.4. Quantification of Prophage Copies in Lysogens

The procedure was based on hybridization of radioactive lysogen DNA (containing prophage) with excess nonradioactive λ DNA as developed by Mandal et al. [12] and is briefly as follows. The bacterial DNA was metabolically labeled with ^3^H-thymidine (4 μCi/mL) in the presence of deoxyadenosine (250 μg/mL). Following 2 h of labeling, the bacteria were lysed by lysozyme-EDTA and the DNA purified by deproteinization with phenol-chloroform [31]. The DNA solution was dialyzed against 0.01 × SSC, pH 7 [31], 1 mM EDTA. Non-labeled DNA was isolated from λ phage particles by similar deproteinization, diluted to 20 μg/mL, boiled, and chilled rapidly. The solution was made to 6 × SSC and the final DNA concentration was made to 2 μg/mL. Five mL of this DNA solution was filtered through an S&S filter, which was then washed with 100 mL of 6 × SSC, and blank filters (without DNA) were also prepared the same way. All filters were placed in scintillation vials, kept at 37 °C overnight and then at 80 °C for 3 h under vacuum. Prior to DNA–DNA hybridization, 1 mL of a solution containing 0.02% Ficoll, 0.02% polyvinylpyrrolidone, 0.02% bovine serum albumin, and 0.01 M Tris in 3 × SSC were added to each vial, which was then tightly capped and placed in a 65 °C water bath for 6 h. The cellular DNA to be hybridized was diluted to 2 μg/mL and sheared by forcing through a hypodermic needle (size 26) 50 times, then boiled for 15 min and quick-chilled. An equal volume of 12 × SSC was then added to reach a concentration of 1 μg/mL DNA in 6 × SSC, from which 0.5 mL was added to each vial. The vials were incubated for 18 h at 65 °C, then each filter was washed with 150 mL of 3 mM Tris-Cl buffer, pH 8.5, dried, and radioactive count was taken by liquid scintillation counting. This count is proportional to the number of prophage genome copies in the bacterial cellular DNA [12,14].

## Figures and Tables

**Table 1:** The inner membrane activities of the polylysogen and nonlysogen.

Bacteria	Activity / Property Assayed ^1^			
	PTS activity ^a^ Total (in frozen-thawed cells)	Amount exposed in saline-washed cells	% of total exposed in saline-washed cells	
Nonlysogen	43.3 ± 3	0.53 ± 0.02	1.20	
Polylysogen	16.6 ± 2	9.70 ± 0.8	60.5	
	Uptake of:			
	^14^C-Glucose	^14^C-Galactose	^3^H-Uridine	
Nonlysogen	3.60 × 10^4^	3.6 × 10^3^	1.93 × 10^4^	
Polylysogen	2.15 × 10^4^ (60%)	2.3 × 10^3^ (63%)	1.00 × 10^4^ (52%)	
	Enzyme activity in membrane fraction			
	NADH oxidase ^b^	FAD-linked LDH ^b^	Dye reductase ^b^	ATPase ^c^
Nonlysogen	26.5 ± 2	8.00 ± 0.2	14.5 ± 1	28.0 ± 1
Polylysogen	19.0 (60.1%)	4.08 (57%)	8.5 (58%)	14.00 (50%)

^1^ Assay methods and enzyme units are presented in Section 4. LDH = lactate dehydrogenase; FAD = flavin adenine dinucleotide; NADH = nicotinamide adenine dinucleotide. ^a^ One unit of phosphoenolpyruvate (PEP)-dependent phosphotransferase enzyme system (PTS) activity was defined as nmole deoxyglucose phosphorylated per mg protein per minute. ^b^ One unit was defined as the amount of protein that could cause an increase of 0.1 O.D. per minute under the assay conditions. ^c^ One unit was defined as the amount of protein that could liberate 1 μg of inorganic phosphate per minute from ATP under the assay conditions.

**Table 2:** Release of periplasmic enzymes from polylysogens by EDTA treatment.

Enzyme	% Total Activity Released by EDTA Treatment ^1^	
	Nonlysogen	Polylysogen
(a) Periplasmic:		
Alkaline phosphatase	1.8	32.0
5′-nucleotidase	8.0	47.0
Acid phosphatase	ND	30.0
(b) Cytoplasmic:		
G6PD	ND	ND
Inorganic pyrophosphatase	9.0	10.0

^1^ EDTA treatment and enzyme assays are described in the text and in Section 4. Total enzyme activity was assayed in the supernatants of fully lysed cells following EDTA (0.5 mM)-lysozyme (400 μg/mL) treatment. ND = Not detectable. The cytoplasmic enzymes were assayed as control under identical conditions.

**Table 3:** Efficiency of plating of T phages on polylysogen and monolysogen.

Phage	Efficiency of Plating, Relative to *E. coli* 594 ^2^	
	Monolysogen 594 (λ^+^)	Polylysogen 594 (λ*N*^-^*c*I^-^)
T4D	0.8	0.2
T3	0.9	1.0
T7	1.0	1.1

^2^ The efficiency of plating on the experimental bacteria was calculated as the ratio of the number of plaques produced on them to those produced on nonlysogen *E. coli* 594, when the same amount of phage was plated on both. T4D is a wild type strain of T4.

**Table 4:** Quantity and carbohydrate composition of polylysogen lipopolysaccharides (LPS). ^1.^

Bacteria	Total LPS (% of Dry Envelope, *w/v*)	Carbohydrate (% of dry LPS, *w/w*)					
		Monosaccharides					
		Total	Rha	GlcN	Glc	Hep	KDO
Nonlysogen	5.0 (100)	31.3	0.92	6.5	11.1	18.6	4.22
Polylysogen	3.6 (72)	32.8	0.97	6.0	11.9	18.45	4.58

^1^ LPS isolation and carbohydrate assays are described in Section 4. The abbreviations are: Rha, rhamnose; GlcN, N-acetyl glucosamine; Glc, glucose; Hep, heptose; KDO, keto-deoxyoctulosonate. Galactose and colitose could not be detected.

**Table 5:** Relative amounts of major outer membrane proteins of nonlysogen and polylysogen. ^1.^

Major Peak#	~Mr/1000	Area of the Peak *		Ratio (b/a)
		Nonlysogen (a)	Polylysogen (b)	
7	57.0	210	235	1.10
9	39.0	141	82	0.58
11	30.0	214	360	1.60
14	18.0	60	123	2.05
15	14.5	435	99	0.22
17	8.0	93	108	1.16

^1^ The major protein bands of the outer envelope, resolved in SDS-PAGE (Figure 5) were quantified by densitometry, and the values in arbitrary units are presented. # The major peaks are numbered as in Figure 6. The ratios of the quantity of each band in the polylysogen (b) and that in the nonlysogen (a) are shown in the last column. *Arbitrary unit.

**Table 6:** Selected properties of the converted, deconverted, and semi-deconverted polylysogens. ^1.^

Bacteria	Colony Size	Cell Length	% AP Leakage	E.O.P. of T4	λDNA Copy
Nonlysogen ^a^	Large	Small rod (1×)	2.4	1.0	0
Deconverted	Large	Small rod (1×)	3.2	0.9	0
Semi-deconverted	Medium	Short filament (3–4×)	12.0	0.48	16 ± 1
Polylysogen	Small	Long filament (14–25×)	32.0	0.2	22 ± 2

^1^ The bacteria are described in detail in the adjoining text. AP = alkaline phosphatase; E.O.P. = efficiency of plating. ^a^ Data of the nonlysogen (*E. coli* 594) are presented for comparison; the cell length and E.O.P. of T4 plating on the nonlysogen were taken as 1, and the numbers of the other isolates were expressed as its ratio. No prophage λDNA could be detected in the nonlysogen and in the fully deconverted bacteria. Cell lengths were measured from Figure 10.

**Table 7:** Properties of polylysogens of the general type 594(λN^-^cI^-^X^-^).

Lysogen	Colony Size	Cell Morphology	PTS Activity (% of 594)	% AP Leakage	E.O.P. of T4 (Fraction of 594)	λDNA Copy
594(λ*N*^-^*c*I^-^)	Small ^a^	Filament, 15×	39.0	32 ± 4	0.20	24 ± 4
594(λ*N*^-^*c*I^-^*R*^-^)	Small	Filament, 15×	34.6	30 ± 3	0.12	22 ± 3
594(λ*N*^-^*c*I^-^*S*^-^)	Small	Filament, 15×	34.4	29 ± 5	0.10	24 ± 4
594(λ*N*^-^*c*I^-^*R*^-^*S*^-^)	Small	Filament, 15×	24.1	32 ± 2	0.13	23 ± 4
594(λ*N*^-^*c*I^-^*O*^-^)	Large	Short filament, 2×	ND	4.9 ± 1	0.65	8 ± 1
594(λ*N*^-^*c*I^-^*P*^-^)	Large	Short filament, 2×	ND	7.8 ± 0.4	0.60	8 ± 1
594	Large	Small rod, 1×	100	1.8 ± 0.1	1.00	0

^a^ Filament lengths are expressed as times of the unit cell (594) length. AP = alkaline phosphatase. Average cell length was determined from Figure 11.

## Abbreviations

ATP	Adenosine triphosphate
cAMP	Cyclic AMP
CFU	Colony-forming unit
DMSO	Dimethyl sulfoxide
DNA	Deoxyribonucleic acid
DOC	Deoxycholate
*E. coli*	*Escherichia coli*
EDTA	Ethylenediaminetetraacetic acid
E.O.P.	Efficiency of plating
FAD	Flavin adenine dinucleotide
Glc	Glucose
GlcN	N-acetyl glucosamine
Hep	Heptose
HPr	Histidine-containing protein (Phosphocarrier protein)
KDO	2-Keto-3-deoxy-octonate
LDH	Lactate dehydrogenase
LPS	Lipopolysaccharide
MTT	3-(4,5-dimethylthiazol-2-yl)-2,5-diphenyltetrazolium bromide)
NADH	Nicotinamide adenine dinucleotide
*O*_L_	Operator, left
*O*_R_	Operator, right
PAGE	Polyacrylamide gel electrophoresis
PEP	Phosphoenolpyruvate
PMS	Phenazine methosulfate
*P*_L_	Promoter, left
pNPP	Para-nitrophenylphosphate
*P*_R_	Promoter, right
PTS	Phosphotransferase enzyme system
Rha	Rhamnose
RNA	Ribonucleic acid
SDS	Sodium dodecyl sulfate
TCA	Trichloroacetic acid
TEMED	Tetramethylethylenediamine
